# Hybrid PET/CT and PET/MR in Coronary Artery Disease: An Update for Clinicians, with Insights into AI-Guided Integration

**DOI:** 10.3390/jcdd12090338

**Published:** 2025-09-03

**Authors:** Francesco Antonio Veneziano, Flavio Angelo Gioia, Francesco Gentile

**Affiliations:** 1 UOC di Cardiologia, Ospedale di Monselice, ULSS 6 Euganea, Via Guglielmo Marconi 19, 35043 Monselice, Italy; 2 UOC di Cardiologia, Università Campus Bio-Medico di Roma, Via Álvaro del Portillo 21, 00128 Rome, Italy; 3 Health Science Interdisciplinary Center, Scuola Superiore Sant’Anna, 56127 Pisa, Italy

**Keywords:** hybrid imaging, PET/CT, PET/MR, coronary artery disease (CAD), myocardial perfusion, coronary plaque characterization, artificial intelligence (AI), myocardial viability, microvascular dysfunction, revascularization guidance

## Abstract

Imaging techniques such as positron emission tomography/computed tomography (PET/CT) and positron emission tomography/magnetic resonance imaging (PET/MR) have emerged as powerful and versatile tools for the comprehensive assessment of coronary artery disease (CAD). By combining anatomical and functional information in a single examination, these modalities offer complementary insights that significantly enhance diagnostic accuracy and support clinical decision-making. This is particularly relevant in complex clinical scenarios, such as multivessel disease, balanced ischemia, or suspected microvascular dysfunction, where conventional imaging may be inconclusive. This review aims to provide clinicians with an up-to-date summary of the principles, technical considerations, and clinical applications of hybrid PET/CT and PET/MR in CAD. Here, we describe how these techniques can improve the evaluation of myocardial perfusion, coronary plaque characteristics, and ischemic burden. Advantages such as improved sensitivity, spatial resolution, and quantification capabilities are discussed alongside limitations including cost, radiation exposure, availability, and workflow challenges. A dedicated focus is given to the emerging role of artificial intelligence (AI), which is increasingly being integrated to optimize image acquisition, fusion processes, and interpretation. AI has the potential to streamline hybrid imaging and promote a more personalized and efficient management of CAD. Finally, we outline future directions in the field, including novel radiotracers, automated quantitative tools, and the expanding use of hybrid imaging to guide patient selection and therapeutic decisions, particularly in revascularization strategies.

## 1. Introduction

CAD remains the leading cause of death worldwide and continues to represent a major challenge for clinical management due to its complex pathophysiology and heterogeneous manifestations [1,2]. Advances in cardiovascular imaging have substantially improved the evaluation of coronary anatomy, myocardial perfusion, and tissue characteristics, supporting both diagnosis and tailored therapeutic strategies. In this context, hybrid imaging techniques such as PET/CT and PET/MR have emerged as valuable tools, combining anatomical and functional information in a single examination [3].

The use of PET/CT is well established for coronary artery calcium scoring and non-invasive angiography, offering high diagnostic accuracy and prognostic value, particularly in intermediate-risk patients [4]. PET/MR provides additional advantages through superior soft tissue contrast, reduced radiation exposure, and advanced motion correction capabilities, enabling the comprehensive assessment of myocardial fibrosis, inflammation, and viability [5]. Furthermore, the integration of AI into hybrid imaging workflows is showing promise in automating image fusion, enhancing motion correction, improving quantification, and supporting risk stratification [6].

Hybrid imaging is increasingly applied to identify vulnerable plaques and microcalcifications using tracers such as 18F-sodium fluoride, providing insights into plaque biology beyond anatomical stenosis [7]. PET/MR, in particular, offers a detailed evaluation of myocardial pathology with potential incremental value in conditions such as myocarditis, sarcoidosis, and cardiac amyloidosis [8]. Despite these advances, cost, availability, and technical complexity still limit the widespread adoption of hybrid imaging in everyday practice [5].

This review provides an updated overview of PET/CT and PET/MR imaging in CAD, focusing on current applications, technical considerations, the emerging role of AI, limitations, and future directions in precision cardiovascular care.

## 2. Principles and Technical Aspects

PET/CT and PET/MR differ primarily in the anatomical modality paired with positron emission tomography, each offering distinct advantages depending on the clinical scenario. PET/CT combines PET with computed tomography, ensuring high spatial resolution, short acquisition times, and widespread availability. It is particularly effective for routine evaluation of coronary artery disease, viability assessment, and detection of inflammatory or infectious conditions. PET/MR, on the other hand, integrates PET with magnetic resonance imaging, which provides superior soft tissue contrast and enables radiation-free multiparametric imaging. This makes it especially useful in younger patients, for longitudinal follow-up, or when detailed tissue characterization is required [9].

Both modalities utilize integrated or co-registered acquisition techniques to align metabolic and structural information with high precision [10]. PET/MR adds further value by offering simultaneous MR sequences such as late gadolinium enhancement and T1/T2 mapping, allowing the assessment of edema, fibrosis, and myocardial tissue composition alongside PET signals.

Commonly used PET tracers include 18F-fluorodeoxyglucose (FDG) for evaluating metabolic activity and inflammation, and perfusion agents like ^13^N-ammonia, ^82^Rb, and ^15^O-water, selected based on institutional logistics and diagnostic objectives. Newer tracers such as Flurpiridaz F18—recently approved by the FDA—and ^68^Ga-FAPI are expanding the diagnostic range of hybrid imaging, supporting the noninvasive evaluation of perfusion, fibroblast activation, and plaque biology [5,11]. In multicenter trials, Flurpiridaz F18 has demonstrated improved sensitivity compared to traditional SPECT agents and allows for exercise-based stress imaging protocols, offering advantages in both image quality and workflow flexibility [11].

Accurate image acquisition requires appropriate patient preparation, particularly when FDG is used for inflammatory imaging, where dietary protocols and pharmacologic interventions are necessary to suppress physiological myocardial uptake. PET/MR supports this process through advanced MR-based motion correction and attenuation correction techniques that enhance quantification of myocardial metabolism and perfusion. The use of Dixon-based sequences, ultrashort echo time imaging, and respiratory or cardiac gating contributes to more consistent co-registration and reduces artifacts in cardiac imaging [3]. In parallel, AI-based methods are being integrated into the workflow, enabling automated co-registration, generation of synthetic attenuation maps, and extraction of quantitative biomarkers that improve consistency and diagnostic performance across platforms [10].

PET/MR is particularly suited for the assessment of myocardial inflammation and post-infarction remodeling, where it enables simultaneous evaluation of PET-derived metabolic activity and MR-derived tissue changes such as fibrosis and edema. This combined information may improve the identification of active disease and guide therapeutic strategies [12].

Hybrid imaging techniques are also being applied to the study of coronary atherosclerosis, where the ability to capture both plaque morphology and molecular activity in a single session offers promising perspectives for risk stratification [13]. These developments continue to shape the evolving role of PET/CT and PET/MR in the context of cardiovascular imaging, supported by ongoing innovation in tracers, acquisition strategies, and computational tools [14].

### Technical Parameters: Spatial/Temporal Resolution, Acquisition Time, and Radiation Dose

Cardiac PET typically achieves a spatial resolution of ~3–5 mm, with temporal resolution determined by the coincidence timing window and framing protocol; ECG-gated acquisitions yield 8–16 frames per cardiac cycle, corresponding to an effective temporal resolution of 50–125 ms. CCTA provides a submillimetric in-plane resolution (~0.3–0.6 mm) and temporal resolution of ~66–175 ms depending on scanner generation [15], while cardiac MR offers an in-plane resolution of ~1.4–2.0 mm (slice thickness 6–8 mm) and temporal resolution of ~30–50 ms (20–30 frames per cardiac cycle) for cine imaging [16].

In hybrid systems, PET/CT integrates PET’s high sensitivity and quantitative perfusion/metabolic assessment with CT’s superior coronary anatomy depiction and rapid attenuation correction. PET/MR combines PET with high soft-tissue contrast, multiparametric tissue characterization (LGE, T1/T2 mapping), and absence of ionizing radiation from the MR component, albeit at the cost of longer acquisition times and greater technical complexity [17].

Typical acquisition times vary; rest–stress PET perfusion studies require ~20–45 min (shorter with ^82^Rb); FDG viability protocols take ~90–120 min including uptake phase; CCTA is completed in seconds (<1 min scan time), though patient preparation and contrast injection extend total table time to ~10 min; comprehensive cardiac MR protocols last ~30–45 min; and integrated PET/MR protocols 45–75 min depending on the MR sequences used [16,18].

Radiation exposure is protocol-dependent; modern prospective-gated CCTA typically delivers 1–5 mSv, low-dose CT for attenuation correction adds ~0.5–1 mSv, and PET perfusion doses are ~1–4 mSv (^15^O-water/^82^Rb/^13^N-ammonia) and ~3–7 mSv for FDG viability imaging. PET/MR eliminates the CT-derived dose, leaving only the PET contribution [15,18].

## 3. Clinical Applications in Ischemic Artery Disease

### 3.1. Ischemia and Viability Assessment

Hybrid PET/CT and PET/MR imaging provide powerful tools for assessing myocardial ischemia and viability in patients with suspected or established CAD. PET perfusion imaging using tracers such as ^13^N-ammonia, ^15^O-water, or ^82^Rb during pharmacologic stress enables the identification of flow-limiting stenoses with high diagnostic sensitivity. Concurrently, metabolic imaging with ^18^F-FDG offers critical insight into myocardial viability, especially in detecting hibernating myocardium that may benefit from revascularization [17].

PET/MR is particularly advantageous for viability assessment due to its ability to simultaneously acquire high-resolution tissue characterization and metabolic data. In this setting, the presence of preserved FDG uptake in regions with reduced perfusion—i.e., a perfusion-metabolism mismatch—has been shown to predict recovery of contractile function after revascularization. In one study, nearly 80% of such mismatched segments regained systolic function post-intervention [19]. This approach aids in stratifying patients who are most likely to benefit from coronary intervention, minimizing unnecessary procedures. The addition of cardiac MR components, including late gadolinium enhancement (LGE), T1 mapping, and myocardial strain imaging, further refines the assessment of scar burden and residual viability. When paired with PET findings, this comprehensive evaluation improves prediction of functional recovery [20].

Recent data support the prognostic relevance of hybrid imaging-derived markers. In a study of over 700 patients, the extent of viable but hypoperfused myocardium was associated with improved event-free survival when revascularization was performed [21]. Furthermore, quantitative flow measures derived from PET, including myocardial flow reserve and stress perfusion, have been shown to outperform angiographic severity in predicting adverse outcomes, as highlighted in population-level analyses [22].

By integrating anatomical, perfusional, and metabolic parameters, hybrid imaging offers a robust platform for identifying jeopardized but salvageable myocardium and guiding personalized therapeutic decisions in ischemic heart disease

### 3.2. Anatomy–Function Mismatch and Microvascular Disease

Hybrid PET/CT and PET/MR have redefined the evaluation of coronary physiology by enabling precise quantification of myocardial blood flow (MBF) and coronary flow reserve (CFR), even in the absence of significant epicardial stenoses. This allows clinicians to detect functional impairments that would otherwise go unnoticed. Anatomical–functional mismatch—where coronary CT angiography appears normal but PET reveals perfusion abnormalities—is often a marker of coronary microvascular dysfunction (CMD) or diffuse atherosclerosis. This mismatch carries independent prognostic value and plays a key role in guiding clinical management [22].

Among the most authoritative frameworks for CMD classification, the expert consensus by Schindler et al. has established a standardized nomenclature distinguishing between “classical” CMD, characterized by impaired hyperemic MBF, and “endogen” CMD, defined by increased resting MBF with preserved hyperemia. This classification, endorsed by the Society of Nuclear Medicine and Molecular Imaging, is based on PET-derived thresholds such as myocardial flow reserve (MFR) <2.0 or <1.7, and is now recommended for clinical reporting and therapeutic guidance [23]. Importantly, CMD—particularly in the endogen form—has been associated with elevated sympathetic tone, hypertension, and metabolic syndrome, and may account for symptoms in patients with non-obstructive coronaries. These patients often present with persistent angina despite normal angiograms, a clinical scenario that is increasingly recognized in women, diabetics, and individuals with obesity or hypertrophic remodeling [23].

PET imaging is uniquely suited for capturing the subtleties of CMD. It allows the quantitative assessment of both global and regional MBF and CFR, offering a pathophysiological window into coronary circulation that goes beyond luminal narrowing. PET/MR extends this further by integrating functional, structural, and inflammatory data, thereby improving diagnostic accuracy in patients with INOCA (ischemia and non-obstructive coronary artery disease). Notably, PET findings often diverge from anatomical imaging, particularly in women and diabetics, as shown in recent outcome studies [24]. A growing body of evidence supports the idea that these discrepancies are not incidental, but reflect underlying microvascular pathology, as also demonstrated in hybrid PET/MR protocols [25].

Moreover, PET enables the subclassification of microvascular angina into meaningful phenotypes. The “classical” type typically involves hyperemic flow impairment, whereas the “endogen” form reflects elevated resting MBF due to increased myocardial workload, as seen in obesity, hypertensive heart disease, or sympathetic overdrive. This phenotyping provides a framework for targeted therapeutic strategies. In the “classical” CMD, vasodilators or anti-anginal agents may be preferred, while the “endogen” variant may benefit from neurohumoral modulation with beta-blockers, ACE inhibitors, or lifestyle modifications. The importance of this functional stratification is supported by data showing that over 50% of patients with angina and normal coronaries actually have CMD, highlighting the need for noninvasive physiological assessment in daily practice [26]. As demonstrated by Tonet et al. [27], PET-derived flow metrics have diagnostic and prognostic value in conditions such as hypertrophic cardiomyopathy and aortic stenosis, where impaired perfusion may precede structural myocardial changes and contribute to symptom burden.

In these settings, PET/CT typically achieves a reconstructed spatial resolution of ~3–4 mm, while PET/MR reaches ~4–5 mm but benefits from superior soft-tissue contrast for tissue characterization [16,17]. Both modalities may be affected by non-ischemic ^18^F-FDG uptake related to systemic inflammatory, infectious, or immunologic disorders, which can mimic ischemia or microvascular dysfunction. Careful patient preparation and correlation with MR tissue markers such as edema or late gadolinium enhancement can help distinguish true ischemic findings from inflammatory artifacts [15,18].

### 3.3. Revascularization Guidance and Prognostic Value

The integration of hybrid imaging into clinical decision-making has meaningfully transformed revascularization strategies in coronary artery disease. By combining detailed anatomical visualization with robust functional quantification, PET/CT and PET/MR enable refined patient selection—discriminating those who are likely to benefit from invasive procedures from those who may be managed conservatively. Revascularization guided by PET-derived ischemic burden and flow reserve has been associated with better outcomes compared to approaches relying on anatomy alone [5].

In the setting of multivessel disease, PET plays a crucial role in unmasking balanced ischemia, helping to avoid false-negative results that may occur with relative perfusion techniques. Quantitative flow imaging allows clinicians to map perfusion deficits to specific coronary territories and to assess the hemodynamic relevance of each lesion. This is particularly valuable when standard stress tests provide inconclusive results or when discordance exists between symptoms and anatomical findings [26].

PET/MR further enhances this approach by enabling simultaneous evaluation of myocardial perfusion, fibrosis, wall motion, and strain. This integration supports complex decision-making in patients with borderline lesions, prior infarction, or left ventricular dysfunction [28]. In chronic coronary syndromes, hybrid imaging improves the detection of hibernating myocardium—regions with impaired flow but preserved viability—that might be missed by conventional imaging techniques.

Prognostically, PET-derived myocardial flow reserve (MFR) has emerged as a robust predictor of outcomes. An MFR below 1.5 has been linked to a markedly increased risk of cardiovascular events, even in the absence of obstructive epicardial disease [26]. Beyond global MFR, regional stress myocardial blood flow and flow capacity are increasingly used to guide vessel-level decisions. As shown by Hoek et al. [29], stress MBF and coronary flow capacity not only predict ischemic burden but can also inform the magnitude of functional recovery after revascularization. Their work highlights that regions with severely reduced flow capacity demonstrate significant improvement in perfusion and clinical status following intervention, while territories with preserved baseline flow show minimal benefit. This supports a shift toward physiology-guided, vessel-specific planning.

Importantly, outcomes appear most favorable when clinical decisions align with PET findings. In contrast, discordance between imaging-derived recommendations and actual treatment strategy has been associated with worse prognosis in multiple trial sub-analyses. This reinforces the role of hybrid imaging not only in diagnostic assessment but as a central tool for therapeutic planning.

Another emerging advantage of PET/MR lies in its potential to reduce overtreatment. By integrating functional and structural information—including markers of irreversible myocardial damage—it allows for more selective revascularization, avoiding procedures in nonviable territories [30].

### 3.4. Plaque Biology and Risk Stratification

Hybrid PET/CT and PET/MR imaging are increasingly applied to assess plaque vulnerability by detecting biological activity beyond luminal stenosis. Among available tracers, ^18^F-sodium fluoride (NaF) PET has emerged as a marker of active microcalcification, reflecting early mineral deposition and ongoing plaque remodeling. This signal, typically localized at the fibrous cap, correlates with biomechanical stress and risk of rupture, particularly in the presence of persistent inflammatory activity [28]. In contrast to static coronary calcium scoring, ^18^F-NaF uptake captures dynamic processes and allows in vivo identification of high-risk lesions before overt structural changes become visible on CT.

Prospective studies have shown that ^18^F-NaF PET/CT can accurately differentiate culprit from non-culprit plaques in patients with recent acute coronary syndromes. Tracer uptake intensity has been associated with subsequent cardiovascular events, independently of conventional risk factors and calcium burden. Importantly, ^18^F-NaF has demonstrated the ability to identify metabolically active plaques even in patients with low or intermediate calcium scores, supporting its added value in refining individual prognostic assessment [31]. Moreover, focal areas of increased uptake often do not match angiographic severity, further emphasizing its role in detecting biologically vulnerable lesions that may be underestimated by anatomy-based methods.

When integrated with coronary CTA, hybrid imaging enhances spatial localization and provides complementary information on lesion characteristics. The association between ^18^F-NaF signal and adverse morphological features—such as positive remodeling, low-attenuation plaque, and spotty calcification—has been shown to improve the identification of plaques at risk of progression or rupture [32]. Advances in image acquisition, motion correction, and quantification pipelines are further contributing to the reproducibility and clinical applicability of this approach.

PET/MR offers the additional advantage of simultaneously evaluating structural, metabolic, and inflammatory components of plaque instability within a single session. Early investigations suggest that high-resolution plaque characterization with PET/MR, especially when combined with machine learning algorithms, could support patient-level risk prediction and monitoring in stable coronary syndromes [13]. In this context, recent work has also emphasized how advanced imaging modalities, including PET and CMR, may help stratify the arrhythmic and ischemic risk of cardiovascular patients by capturing the underlying myocardial substrate and autonomic profile, integrating functional and anatomical data for more precise risk modeling [33].

### 3.5. Inflammatory and Infiltrative Myocardial Disease in CAD Context

Beyond ischemia, hybrid imaging techniques such as PET/CT and PET/MR are playing an increasingly important role in evaluating inflammatory and infiltrative myocardial conditions that may coexist with or mimic coronary artery disease. In particular, ^18^F-FDG PET enables in vivo detection of active inflammation and has proven highly valuable in the diagnosis and monitoring of cardiac sarcoidosis, where focal myocardial uptake correlates with arrhythmic risk and worse outcomes [32]. Similarly, in myocarditis, PET can reveal persistent metabolic activity even after normalization of serum markers or ventricular function, offering a sensitive tool for longitudinal follow-up [34]. Recent data have shown that FDG uptake may remain detectable despite apparent clinical recovery, supporting its role as a non-invasive marker of residual disease activity. In this setting, hybrid PET/MR can provide complementary information on tissue edema and fibrosis and guide decisions regarding the duration or escalation of immunosuppressive therapy. PET imaging has thus gained relevance not only in diagnosis but also in longitudinal risk stratification, especially in patients with recurrent symptoms or at risk of arrhythmias [34].

The integration of PET with MR adds further value by providing high-resolution structural and tissue characterization. In post-infarction remodeling, for example, PET/MR can help differentiate between scar, residual inflammation, and viable tissue, with significant implications for prognosis and therapeutic planning [28].

**Figure 1:**
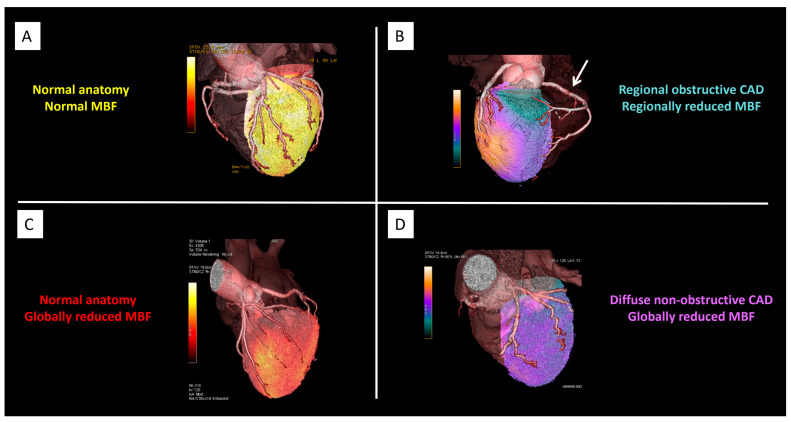
Hybrid PET/CT and PET/MR in CAD. Representative cases illustrating different pathophysiological patterns in CAD as assessed by hybrid imaging. Panel (**A**): Normal coronary anatomy with preserved myocardial blood flow (MBF). Panel (**B**): Regional obstructive CAD with concordant regional reduction in MBF (arrow). Panel (**C**): Normal coronary anatomy but globally reduced MBF, consistent with diffuse microvascular dysfunction. Panel (**D**): Diffuse non-obstructive CAD with globally reduced MBF and abnormal flow distribution. Abbreviations: PET = positron emission tomography; CT = computed tomography; MR = magnetic resonance; CAD = coronary artery disease; MBF = myocardial blood flow. Courtesy of Courtesy of Morrone D, Gentile F, Aimo A, et al. [38].

Hybrid imaging is also emerging as a promising tool in infiltrative cardiomyopathies such as cardiac amyloidosis. PET tracers originally developed for neurodegenerative disorders—like ^11^C-PiB or ^18^F-florbetaben—have demonstrated myocardial uptake consistent with amyloid deposition, suggesting a role for PET/MR in non-invasive diagnosis and disease quantification [35]. Finally, the development of novel tracers such as ^68^Ga-DOTATATE and the establishment of standardized protocols across the “4Is” (inflammatory, infective, infiltrative, and innervation-related diseases) by nuclear and cardiovascular imaging societies support broader adoption of hybrid imaging in clinical practice, ensuring consistency and diagnostic accuracy [36], as also supported by emerging evidence on diffuse low-grade myocardial inflammation in non-infarcted regions after acute ischemia [37]. Figure 1 provides an illustrative example of how hybrid PET/CT and PET/MR can be applied in the clinical setting to characterize different pathophysiological patterns of CAD.

### 3.6. Modality Selection and Complementary Use

Choosing between PET/CT and PET/MR depends on the primary clinical question, patient characteristics, and local resources. PET/CT is generally preferred when high-quality coronary anatomy is required—such as for stenosis evaluation, coronary calcium scoring, or plaque characterization—especially if stents or heavy calcifications are present. It also offers rapid acquisition, broad availability, and integration with coronary CTA, making it suitable for acute or time-sensitive assessments [3,4].

PET/MR is favored when radiation minimization is important (e.g., in younger patients or those requiring serial follow-up) and when multiparametric tissue characterization is central to the diagnosis, such as in myocarditis, sarcoidosis, or infiltrative cardiomyopathies. It also provides advanced motion correction and superior soft-tissue contrast, making it particularly valuable for combined ischemia and viability assessment or when inflammatory and ischemic pathologies coexist [5,32].

Combined use of PET/CT and PET/MR may be warranted in selected complex scenarios. When anatomy is the main uncertainty—such as suspected multivessel disease or ambiguous stenoses—PET/CT with CTA is typically performed first, followed by PET/MR if detailed tissue characterization is needed. Conversely, when myocardial substrate evaluation is the primary goal—such as suspected inflammatory disease—PET/MR may be performed first, with PET/CT reserved for detailed anatomical clarification. This sequential approach maximizes diagnostic yield while balancing radiation exposure and resource use [18].

## 4. Limitations of Hybrid Imaging in CAD

While hybrid PET/CT and PET/MR imaging have shown considerable promise in the evaluation of CAD, their clinical integration remains limited by several important constraints. Among the foremost, cost and limited accessibility continue to hinder widespread use—particularly for PET/MR systems, which are less prevalent due to high acquisition and maintenance costs, infrastructure demands, and the need for specialized staff and dedicated workflows [39]. Although PET/CT is more routinely implemented in clinical practice, it still poses logistical challenges including dual-modality scheduling, strict patient preparation protocols (especially for myocardial ^18^F-FDG suppression), and the need to mitigate extracardiac tracer uptake, which can compromise diagnostic quality [40].

From a technical standpoint, each modality carries specific limitations. PET/MR, for instance, offers inferior spatial resolution for coronary imaging compared to CT, and is more susceptible to motion-related artifacts and image misregistration—especially in patients with arrhythmias, rapid breathing, or limited compliance. While recent developments in motion correction algorithms and attenuation correction methods (e.g., Dixon-based sequences) are promising, their incorporation into standardized clinical practice is still evolving [40]. Quantitative parameters such as myocardial blood flow or standardized uptake values (SUVs) remain variably defined across centers and vendors, limiting reproducibility in multicenter trials and real-world comparisons. Radiation exposure, though significantly minimized with contemporary PET/CT protocols, remains a concern in young or high-risk patients requiring serial studies. PET/MR, in contrast, eliminates radiation from the anatomic component, making it particularly attractive in pediatric, adolescent, or oncology patients [41]. However, its use is limited by longer acquisition times, patient discomfort, and reduced scanner availability, all of which affect patient throughput and broader applicability [42]. Moreover, although hybrid imaging provides valuable functional and pathophysiological insights, current evidence remains insufficient to support its impact on clinical outcomes. The absence of large-scale randomized controlled trials demonstrating superiority over conventional diagnostic algorithms restricts its incorporation into guidelines and reimbursement systems, and may limit adoption outside of research settings or highly specialized centers [43].

An additional consideration concerns the impact of spatial and temporal resolution, acquisition time, and radiation exposure. PET typically provides spatial resolution around 3–5 mm and temporal resolution of 50–125 ms (with ECG gating). CT achieves ~0.3–0.6 mm resolution and ~66–175 ms temporal resolution, while MR offers ~1.4–2.0 mm spatial and ~30–50 ms temporal resolution. These factors influence perfusion quantification, motion correction, and tissue characterization. PET/CT ensures rapid acquisition but involves radiation exposure (1–5 mSv for CCTA plus PET dose), whereas PET/MR avoids CT-derived radiation but requires longer scan times (up to 75 min) and higher patient compliance. Interference from systemic inflammatory or immunologic conditions may also impair image interpretation, particularly with FDG [3,15,16,18].

Ultimately, unlocking the full clinical potential of hybrid imaging will require a multifaceted effort: technological refinement, harmonization of protocols, cost-effectiveness analyses, and above all, prospective outcome data demonstrating therapeutic impact. It will also be essential to consider patient-centered factors such as tolerability, scan duration, and availability when integrating these techniques into care pathways. In this evolving landscape, PET/MR has shown that many of its initial technical challenges—such as attenuation correction, motion compensation, and spatial resolution—are being actively addressed, with increasingly promising performance in soft tissue imaging, neurology, and specific oncologic or pediatric indications [43].

A summary of key clinical applications of hybrid PET/CT and PET/MR imaging in the context of coronary artery disease is presented in Table 1.

## 5. Future Directions and Artificial Intelligence

The future of hybrid imaging in CAD is closely tied to the ongoing advances in AI, which is progressively transforming both image acquisition and post-processing interpretation. Deep learning algorithms, in particular, have demonstrated significant potential in PET image denoising and reconstruction, enabling not only shorter acquisition protocols but also the reduction in administered radiotracer doses. This translates into improved patient safety and optimized workflow efficiency [6]. Notably, AI-based reconstruction techniques have enabled up to 75% dose reduction in FDG-PET imaging without compromising diagnostic accuracy, a development that represents a substantial advancement in clinical practice [44]. Beyond image generation, AI is redefining the analytical dimension of hybrid imaging. Automated segmentation tools powered by convolutional and recurrent neural networks are being increasingly employed to extract quantitative parameters such as myocardial perfusion, ejection fraction, and wall motion from hybrid MPI datasets. These tools have shown diagnostic performance comparable to experienced human readers, particularly in the identification of perfusion defects and the prediction of obstructive CAD. For instance, a deep learning model trained on stress polar maps alone achieved an AUC of 0.84 in detecting obstructive disease, underscoring the efficacy of AI as a non-invasive diagnostic adjunct [45]. Emerging hybrid AI frameworks are now integrating imaging features with clinical, biochemical, and demographic variables to support more accurate risk stratification. Machine learning classifiers—such as support vector machines, random forests, and gradient boosting—are being used to merge anatomical and functional imaging data with patient-specific variables. These models enhance outcome prediction, particularly in intermediate-risk populations, and hold promise for informing personalized therapeutic strategies in complex clinical scenarios [46]. Moreover, deep learning is streamlining the quantification of cardiac function and hemodynamics using advanced modalities such as 4D flow MRI and dynamic PET perfusion imaging. These techniques, previously limited by operator dependence and time constraints, are now being automated through AI to deliver reproducible measures of coronary flow reserve, ejection fraction, and regional wall motion in a standardized manner [45]. Such operator-independent quantification is essential for harmonizing results across centers and improving diagnostic reliability. Recent evidence further supports the integration of holistic, AI-enhanced hybrid imaging for prognostic applications.

In a large multicenter study of over 10,000 patients, Marcinkiewicz et al. [47] demonstrated that a fully automated AI model incorporating myocardial perfusion SPECT, CT attenuation correction (CTAC), coronary calcium score (CAC), epicardial adipose tissue (EAT), and radiomic features from extracardiac structures significantly outperformed conventional models in predicting all-cause mortality (AUC 0.80). Similarly, Miller et al. showed that AI-derived cardiac chamber volumes and left ventricular mass obtained from CTAC scans improved risk stratification when combined with perfusion imaging, particularly by adding information not available in MPI alone, such as left atrial and right ventricular volumes. These variables were independently associated with major adverse cardiovascular events and contributed to a 23% improvement in continuous net reclassification.

An additional layer of innovation comes from the hybridization of machine learning and classical survival analysis. Juarez-Orozco et al. proposed an integrated approach in which ML-derived scores from PET and coronary CT angiography were input into Cox proportional hazard models, significantly improving long-term prediction of myocardial infarction and mortality. This methodology combines the high-dimensional capacity of machine learning with the interpretability and time-to-event sensitivity of traditional biostatistics—offering a viable and explainable framework for clinical adoption [48]. Lastly, explainable AI (XAI) frameworks are being actively developed to foster clinician trust and transparency in model outputs. By employing methods such as SHAP (SHapley Additive exPlanations) and feature importance analysis, AI models can now provide interpretable justifications for their predictions, helping bridge the gap between computational inference and clinical decision-making [49,50,51]. Looking ahead, artificial intelligence is poised to become a central enabler of hybrid imaging, not by replacing clinical expertise, but by amplifying its scope and precision. Through the automation of complex tasks, enhancement of reproducibility, and integration of multi-modal data, AI has the potential to democratize access to high-quality cardiovascular imaging. As external validations increase and regulatory pathways mature, AI-powered PET/CT and PET/MR are expected to play a pivotal role in advancing precision cardiovascular medicine [47,49].

A selection of the most relevant clinical applications of artificial intelligence in hybrid PET/CT and PET/MR imaging is summarized in Table 2.

## 6. Conclusions

Hybrid PET/CT and PET/MR imaging have emerged as powerful tools in the evaluation of coronary artery disease, enabling the simultaneous assessment of anatomical, functional, and metabolic parameters. These modalities improve diagnostic accuracy and clinical decision-making, especially in complex cases such as microvascular dysfunction, multivessel disease, and inflammatory cardiomyopathies.

**Figure 2:**
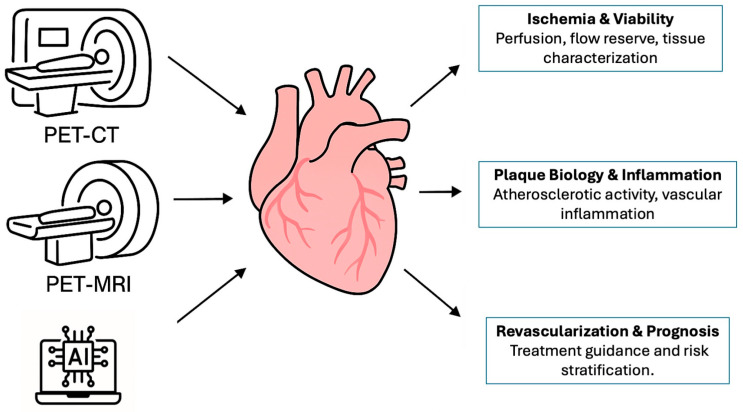
AI-supported hybrid imaging in coronary artery disease: PET/CT and PET/MR enable functional and anatomical assessment, enhanced by artificial intelligence. Abbreviations: AI = artificial intelligence; PET-CT = positron emission tomography/computed tomography; PET-MRI = positron emission tomography/magnetic resonance imaging.

The integration of artificial intelligence further enhances the potential of hybrid imaging, enabling automated quantification, image reconstruction, and risk prediction. While current limitations—such as cost, accessibility, and lack of outcome-based validation—still restrict widespread adoption, ongoing advances in technology, AI applications, and novel tracers are rapidly addressing these barriers. Ultimately, hybrid imaging represents a key step toward precision cardiology, with the potential to support more personalized, efficient, and biologically guided management of coronary artery disease. The combined use of PET/CT, PET/MR, and artificial intelligence enables a comprehensive management of coronary artery disease, as illustrated in Figure 2.

## Figures and Tables

**Table 1:** Clinical applications of hybrid PET/CT and PET/MR imaging in coronary artery disease.

Application	Description	Reference	Preferred Modality/Rationale
Ischemia assessment	Quantitative perfusion imaging using ^13^N-ammonia, ^15^O-water or ^82^Rb during stress testing	[4,15,16,18]	**Either**: PET/CT for rapid workflow and coronary anatomy; PET/MR if tissue characterization or radiation minimization is important
Viability imaging	FDG uptake combined with MR markers (LGE, T1 mapping) to assess hibernating myocardium	[16,17,18]	**PET/MR preferred** LGE, mapping ± FDG; PET/CT if MR is contraindicated
Coronary microvascular dysfunction (CMD)	Evaluation of myocardial flow reserve (MFR); CMD classification (classical vs. endogen types)	[20,21,22,23]	**Either**: PET/MR adds tissue characterization, PET/CT provides faster acquisition
Vulnerable plaque detection	Identification of biologically active plaques with ^18^F-NaF PET uptake	[7,30,31]	**PET/CT preferred** for co-localization of NaF uptake with CTA high-risk features
Infiltrative and inflammatory cardiomyopathies	Combined FDG-PET and MR for sarcoidosis, myocarditis, amyloidosis in CAD contexts	[34,35,36]	**PET/MR preferred** for comprehensive tissue characterization without radiation
Revascularization planning	Integration of perfusion and viability data to guide interventions and avoid unnecessary PCI	[23,24,25]	**Either**: PET/CT if CTA needed for vessel planning, PET/MR if viability and scar burden assessment is key
AI-assisted quantification and prediction	Deep learning for segmentation, risk stratification, and ultra-low-dose PET	[14,42]	**Either**, depending on base modality; AI enhances both

Abbreviations: PET = positron emission tomography; CT = computed tomography; MR = magnetic resonance; CAD = coronary artery disease; CMD = coronary microvascular dysfunction; MFR = myocardial flow reserve; LGE = late gadolinium enhancement; FDG = fluorodeoxyglucose; NaF = sodium fluoride; PCI = percutaneous coronary intervention; AI = artificial intelligence.

**Table 2:** Selected AI applications in hybrid PET/CT and PET/MR imaging for CAD.

AI Application	Description	References
**Dose reduction and image reconstruction**	Deep learning enables PET image denoising and reconstruction with up to 75% dose reduction, preserving diagnostic accuracy and improving efficiency.	[44]
**Automated functional analysis**	AI extracts myocardial perfusion, ejection fraction, and wall motion from MPI datasets, achieving expert-level diagnostic performance.	[16,46]
**Advanced risk stratification**	ML integrates imaging, clinical, and biochemical variables for individualized risk prediction in CAD patients.	[47,48]
**Multimodal prognostic modeling**	Hybrid AI models combining CTAC, CAC, EAT, and radiomics enhance prediction of MACE and mortality across large populations.	[48]
**Explainable and interpretable AI (XAI)**	SHAP and feature attribution methods provide transparent model reasoning, bridging AI output with clinical decision-making.	[49,50]

Abbreviations: AI = artificial intelligence; CAD = coronary artery disease; PET = positron emission tomography; CT = computed tomography; MR = magnetic resonance; MPI = myocardial perfusion imaging; CTAC = CT attenuation correction; CAC = coronary artery calcium; EAT = epicardial adipose tissue; MACE = major adverse cardiovascular events; XAI = explainable artificial intelligence; SHAP = SHapley Additive exPlanations.

## Data Availability

The original contributions presented in the study are included in the article. Further inquiries can be directed to the corresponding author.

## Abbreviations

The following abbreviations are used in this manuscript:

CAD	Coronary Artery Disease
PET	Positron Emission Tomography
CT	Computed Tomography
MR	Magnetic Resonance
PET/CT	Positron Emission Tomography/Computed Tomography
PET/MR	Positron Emission Tomography/Magnetic Resonance Imaging
FDG	Fluorodeoxyglucose
NaF	Sodium Fluoride
CFR	Coronary Flow Reserve
MBF	Myocardial Blood Flow
MFR	Myocardial Flow Reserve
AI	Artificial Intelligence
CMR	Cardiac Magnetic Resonance
CMD	Coronary Microvascular Dysfunction
MPI	Myocardial Perfusion Imaging
LGE	Late Gadolinium Enhancement
CTAC	CT Attenuation Correction
CAC	Coronary Artery Calcium
EAT	Epicardial Adipose Tissue
XAI	Explainable Artificial Intelligence
SHAP	SHapley Additive exPlanations
APC	Article Processing Charge
